# The SDF-1**α**3′A Genetic Variation Is Correlated with Susceptibility of Asthma in Iranian Patients

**DOI:** 10.1155/2013/759361

**Published:** 2013-08-20

**Authors:** Houshang Rafatpanah, Masoud Amin, Mohsen Ghasemshirazi, Mohammad Kazemiarababadi, Hossein Khorramdelazad, Hamid Abousaidi, Ziba Shabani, Ahmadreza Sayadi, Gholamhossein Hassanshahi, Jamile Samadi

**Affiliations:** ^1^Immunology Research Center, School of Medicine, Mashhad University of Medical Sciences, Mashhad, Iran; ^2^Molecular Medicine Research Center, Rafsanjan University of Medical Sciences, Rafsanjan 77175-835, Iran; ^3^Physiology, Pharmacology Research Center, Rafsanjan University of Medical Sciences, Rafsanjan 77175-835, Iran; ^4^Department of Immunology, Rafsanjan University of Medical Sciences, Rafsanjan 77175-835, Iran; ^5^Department of Infectious Diseases, Rafsanjan University of Medical Sciences, Rafsanjan 77175-835, Iran; ^6^Faculty of Nursing, Rafsanjan University of Medical Sciences, Rafsanjan 77175-835, Iran; ^7^Kerman University of Medical Sciences, Kerman, Iran

## Abstract

*Background and Aim.* Chemokine/receptor axis is a predominant actor of clinical disorders. They are key factors of pathogenesis of almost all clinical situations including asthma. Correspondingly, CXCL12 is involved in the immune responses. Therefore, this study was designed to explore the association between gene polymorphism at position +801 of CXCL12, known as SDF-1*α*3′A, and susceptibility to asthma in Iranian patients. *Material and Methods.* In this experimental study, samples were taken from 162 asthma patients and 189 healthy controls on EDTA. DNA was extracted and analyzed for CXCL12 polymorphisms using PCR-RLFP. The demographic information was also collected in parallel with the experimental part of the study by a questionnaire which was designed specifically for this study. *Findings.* Our results indicated a significant difference (*P* < 0.0001) between the *A/A*, *A/G*, and *G/G* genotypes and *A* and *G* alleles of polymorphisms at position +801 of CXCL12. We also showed an elevated level of CXCL12 circulating level in Iranian asthma patients. *Conclusion.* Our findings suggest that SDF-1*α*3′A (CXCL12) polymorphism plays a role in pathogenesis of asthma. It can also be concluded that circulatory level of CXCL12 presumably can be used as one of the pivotal biological markers in diagnosis of asthma.

## 1. Introduction

Asthma is characterized as the airways chronic inflammatory disease with mostly airway obstruction, which is accompanied by increased bronchial high responsiveness to various external and internal stimuli. Asthmatic patients suffer from varying symptoms from breathlessness, wheezing, and cough to chest tightness. It is sometimes coupled with allergy, an acquired potency for progression of unwanted reactions that are controlled via immunological mediators [1]. Approximately more than 22 million people are suffering from asthma. Asthma and allergic diseases are believed to follow the pattern of genetic diseases that are caused by the interaction of multiple genes and also environmental stimuli [2]. 

Although several lines of evidence have indicated the central role of chemokines in regulating multiple aspects of the asthmatic response, the full picture of chemokines' involvement in allergic airway inflammation, the exact role of specific chemokines at various stages in the evolution of allergic lung inflammation and their regulatory pathways in vivo are only partially identified. 

In the process of allergic airway disease, at least one part of diagnosis is the infiltration of blood leukocytes, including eosinophils, neutrophils, lymphocytes, and macrophages in the lung [3]. The CD4+ Th2-type lymphocytes (Th2 cells) and other inflammatory leukocytes in airways mucosa secret a wide range of inflammatory mediators that participate both directly and indirectly in remodeling of the airway wall, excessive mucus secretion, airway obstruction, and finally airway hyperreactivity [4–6].

CXCL12 as a homeostatic CXC chemokine is widely expressed with a broad range of functions (from recruitment of mature B and T cells to migration of haematopoietic progenitor cells from the bone marrow). It is well documented that CXCL12 plays a wide variety of roles in two unrelated set of diseases such as human immunodeficiency virus (HIV) and cancer, because its known receptor (CXCR4) is a coreceptor used by HIV T-tropic strains, and it is also the most widely expressed chemokine receptor in many different types of cancers and asthma [7, 8]. “The SDF-1 gene is located on human chromosome 10q11.1 and SDF1-3′A polymorphism is a single nucleotide polymorphism (SNP) at position 801 relative to the start codon in the 3′ untranslated region involving a G > A transition in SDF-1. The A allele is probably a target of cis-acting factors, and it is assumed to upregulate the expression of SDF-1” [9]. This polymorphism is the best-studied polymorphism in CXCL12 gene [1]. 

The association between this polymorphism and a variety of diseases from lung and breast cancer, acute myeloid lymphoma, leukemia, and chronic myeloproliferative disease to viral infections such as acute hepatitis is well documented [1].

There are no reports describing any association between SDF-1*α* 3′A polymorphism and pathogenesis of asthma. Therefore, the authors of this study hypothesized that this polymorphism may play a critical role in development of asthma in Iranian population; thus, this study investigated the predicted role of the +801G/A polymorphism in pathogenesis and progression of asthma in Iranian population.

## 2. Material and Methods

### 2.1. Subjects

In this study, we recruited 162 unrelated asthmatic patients with a mean age of 51 ± 11 years (ranged between 15 and 79 years) using easy convenience method. Women being pregnant, patients having a history of inflammatory and infectious diseases rather than asthma, and patients with a history of cigarette smoking were excluded from the study. The occurrence of asthma was diagnosed by an expert infectious diseases specialist according to the American Thoracic Society (ATS) criteria. According to the clinical findings and history, patients were classified into 2 groups, that is, allergic and nonallergic groups. We also enrolled 189 genetically unrelated subjects having normal spirometric values and no respiratory symptoms which were matched for sex and similar ethnicity origin with the patients. The study protocol was approved by the ethical committee at our institution (Rafsanjan University of Medical Sciences), and written informed consent was obtained from all participants, either patients or controls, prior to sample collection. Some characteristics of the subjects are summarized in Table 1.

### 2.2. DNA Extraction

Asthmatic and control subjects' peripheral blood samples were collected on Ethylenediaminetetraacetic acid (EDTA) precoated tubes and subjected to genomic DNA extraction using a commercial kit (Bioneer, South Korea). The extracted DNA samples were then stored at −20°C for further use.

### 2.3. Polymorphism Detection 

The CXCL12 gene polymorphism at the position +801 was analyzed by polymerase chain reaction-restriction length polymorphism (PCR-RFLP) method. As previously described by Hassanshahi et al., Azin et al., and Ryabov et al. [10–12], a PCR reaction mixture was made up by addition of the following reagents to a 0.2 mL microcentrifuge tube on ice: 

2.5 *μ*L of *taq* DNA polymerase buffer (10X), 0.5 *μ*L of MgCl2 (stock concentration 1.5 mM), 0.5 *μ*L of each dNTP (dATP, dCTP, dGTP, and dTTP) stock concentration of 10 mM), 1 *μ*L of each primer (forward: CAGTCAACCTGGGCAAAGCC and reverse: AGCTTTGGTCCTGAGAGTCC), stock concentration of 25 ng/*μ*L, 1 *μ*L of prepared DNA, and sterile double-distilled water to a final volume of 25 *μ*L. The following program was used for fragment amplification: one cycle of 93°C for 2 min, 93°C for 1 min (denaturation), 1 min at 57°C for annealing of CXCL12, 72°C for 40 sec (elongation) followed by 30 cycles of 93°C for 20 sec, 55°C for 20 sec, and 72°C for 40 sec. Within the last 45 sec of the first stage, approximately 0.3 *μ*L of Taq DNA polymerase was added to the mixture. *The final volume was 20 *μ*L which contains 2 *μ*L of primer mix of forward and reverse, 10 *μ*L of Master mix (dNTP, MgCl*_2_, and* Taq enzyme), 3 *μ*L of DNA, and 5 *μ*L of DNase-free water.* The amplified PCR product of CXCL12 gene covers +801 regions with a molecular size of 302 bp. The Sac-1 (Fermentas, Lithuania) restriction enzyme has only one restriction site on this region, and is capable to digest the fragment and produce two fragments of 202 and 100bp following digestion. In fact, in case of heterozygotic form (A/G), 3 different fragments with 302, 202, and 100 bp are then visible, while in homozygotic form, a 302 bp fragment (without any digestion (A/A)) or two 202 and 100 bp (digesting both alleles (G/G)) was then observed (Table 2 and Figure 2). The digested products were electrophoresed on a 2.5% agarose gel following addition of 4 *μ*L of loading buffer (Cinnagen, Iran) and studied on ChemiDoc model XRS (Bio-Rad, USA) subsequent to ethidium bromide staining.

### 2.4. Chemokine Assay 

The serum level of CXCL12 was measured by ELISA (R&D systems, UK) in patients and healthy controls immediately after blood collection. Assays were performed as per manufacturer's guidelines. The sensitivity of kits was 2 pg/mL, and inter- and intra-assay assessments of reliability of the kit were conducted.

### 2.5. Statistical Analysis

Hardy-Weinberg equilibrium was assessed using genotype data. Allele and genotype frequencies were calculated in patients and healthy controls by direct gene counting. Statistical analysis of the differences between groups was determined by *χ* ^2^, *t*-test, and ANOVA using SPSS software version 17 which power of test was %90. *P* value of less than 0.05 was considered significant.

## 3. Results

Statistical analysis of demographic parameters indicated that the mean age, gender, and socioeconomical status of the participants had no markedly differences, which were as follows: the mean age of patients was 51 ± 11 years and of control group was 48 ± 12 years (*P* = 0.85), and the gender variation of patients was 129 (56.58%) female and 99 (43.42%) male and for control group was 101 (61%) female and 88 (38.6%) male (*P* = 0.9) (Table 1). 

Analysis of the polymorphisms in +801 of CXCL12 by *S*ac-1 restriction enzyme showed that the frequency of *A/A *genotype was 18 (4.3%) in patients and 27 (6.5%) in controls (OR = 2.541, 95% CI = 1.17 − 5.51, *P* < 0.018). Our results also revealed that the frequency of *A/G* genotype was 190 (45.6%) and 60 (14.4%) in patients and controls, respectively (OR = 0.144, 95% CI = 0.77 − 0.268, *P* < 0.0001).

**Figure 1:**
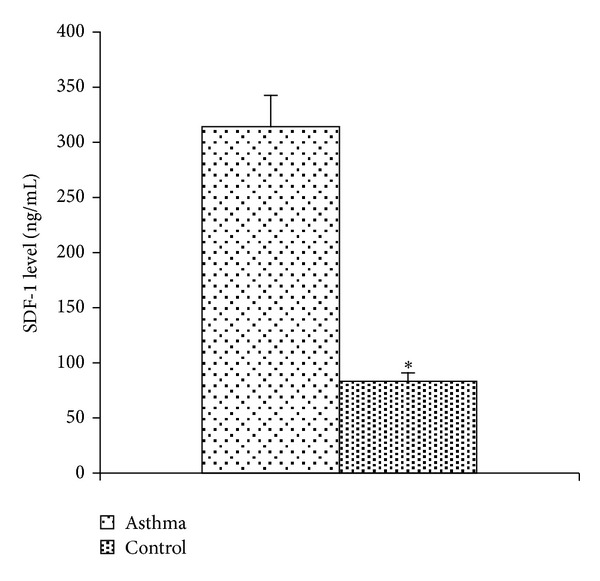
SDF-1*α* (CXCL12) circulating level in asthma patients and controls. ∗= significant difference was observed in SDF-1*α* (CXCL12) serum level (*P* < 0.0001). Data are shown as mean ± SEM.

**Figure 2:**
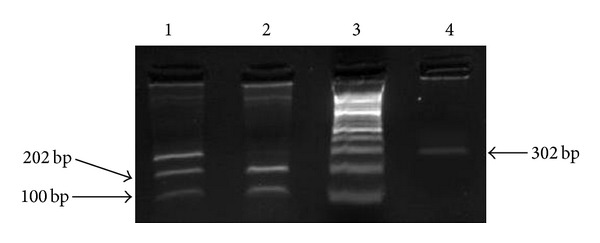
*Sac*-1 digestion of PCR product of SDF-1 gene. Lane 1: heterozygotic digestion of PCR product of SDF-1 (A/G). Lane 2: homozygotic digestion (G/G). Lane 3: 100 bp ladder marker. Lane 4: homozygotic nondigested PCR product (A/A). bp = base pair.

 The frequency of the *G/G* genotype in patients was 20 (4.8%) and in controls was 102 (24.5%) (Table 2), where statistically analyzed data indicated a significant difference between the two groups (*P* < 0.0001) (OR = 4.021, 95% CI = 2.21–7.29, *P* < 0.0001). The frequency of *A* allele was 226 (27.1%) and 114 (13.7%) in patients and controls, respectively. In case of *G* allele, 230 (27.6%) were observed in patients, while the frequency of this allele was 264 (31.7%) in controls (Table 2). Statistical analysis of alleles exhibited a significant difference between patients and controls (*P* < 0.0001). The results of this study also showed that the plasma level of CXCL12 was 312.53 ± 28.75 and 83.57 ± 6.74 pg/mL in asthma patients and healthy controls, respectively (Figure 1). Statistical analysis showed that the difference was significant (*P* < 0.0001). Our results indicated that the protein levels of SDF-1 in AG genotype in asthma and control were 448.75 ± 281 and 171.81 ± 54.11, respectively, which the difference was significant (*P* < 0.0001). Analysis of our data showed that the protein levels of SDF-1 in AA genotype in asthma and control were 22.84 ± 8.41 and 21.62 ± 17.61, respectively, which the difference was not significant (*P* < 0.822). Analysis of data by Eta test did not show significant difference between genotype and protein level of SDF-1 in patient group (approx. sig = 0.654, OR = 11.89/13.61); it also showed that there is no significant difference between FEV1 percent and genotype in patient group (Eta Test, approx. sig = 0.654, OR = 58.40/59.58) and that there is no significant difference between genotype and FEV1/FVC percent in patient group (Eta Test, approx. sig = 0.654, OR = 64.60/65.60). Analysis data by Spearman's test (Spearman's correlation coefficient) showed significant difference between protein level of SDF-1 and FEV1 percent and between protein level of SDF-1 and FEV1/FVC percent in patient group (*P* < 0.0001); this is an inverse relationship between protein level of SDF-1 and FEV1 percent and between protein level of SDF-1 and FEV/FVC percent (Table 3). 

Analysis data with Kruskal-Wallis Test (because this kind of data does not have normal distribution, we had to use this test to obtain mean rank of three genotype polymorphisms for protein level of SDF-1, FEV1 percent, and FEV1/FVC percent) showed that AA, AG, and GG genotypes have the mean rank of 71.38, 46.81, and 56.79 for the protein level of SDF-1, 32.63, 57.19, and 47.21 for the FEV1 percent and 32.63, 57.19, and 47.21 for the FEV1/FVC percent, respectively, in patient group. For all of them, = 0.021, which showed significant difference (*P* < 0.05) for protein level of SDF-1, FEV1 percent, and FEV1/FVC percent (Table 4). 

## 4. Discussion

The current study was undertaken to investigate the role of CXCL12 + 801 G/A SNP polymorphism in susceptibility to asthma in Iranian asthma patients. All of study groups (patients and controls) had same ethnic background origin and shared a common geographic origin in Southeast part of Iran. We demonstrated a closed association between this polymorphism and asthma in Iranian asthma patients. To our best knowledge, this is the first study to report an association between this polymorphism and asthma. The paramount importance of chemokines and their receptors, as the central actors in the initiation and development of asthma, has been evidenced [13, 14]. Results of our study may confirm the role played by SDF-1*α* (CXCL12) angiogenic alterations associated with asthma which was reported by Hoshino et al., 2003 [15].

Hoshino et al. showed that immunoreactivity of CXCL12 is increased within the airways of asthmatic patients and reported that expression of CXCL12 plays an important role in angiogenesis in the airways of asthmatic patients [15]. 

The expanded vascularity that has been reported in the asthmatic mucosa might possibly confirm that angiogenesis is a component of the chronic inflammatory response in asthmatic patients, and increased CXCL12 circulatory level in asthmatics may confirm that angiogenesis is a specific characteristic of asthma, which is probably at least partially mediated by CXCL12 [16–18]. 

However, angiogenesis function of chemokines is currently the focus of intense investigations; little information is known on the roles of chemokines in pathological remodeling of the asthmatic airway. Mice lacking the CXCL12 gene have defective vascular development, suggesting that CXCL12 plays an important role in organ vascularisation [19]. Recent studies have demonstrated that endothelial cells are strongly chemoattracted to CXCL12 [20–22]. Cytokine-mediated regulation of CXCL12 has been demonstrated in eosinophils and lung epithelial cells [23, 24]; the effects of CXCL12/CXCR4 axis showed a contribution to the inflammatory response in a murine model of asthma [25]. Because CXCL12 is a potent chemotactic factor for T and pre-B lymphocytes [26, 27], plasma cells [28], and dendritic cells (DCs) [29] and expression of SDF-1 (CXCL12) receptor (CXCR4) is also documented on eosinophils [24, 30] and mast cells [31–33] - the two critical components in allergic conjunctivitis [33]. It may facilitate infiltration of these cell types to the lung vascular system. CXCL12 may also recruit a wide variety of mature and immature CXCR4 positive leukocytes to the lungs and stimulate neovascularization [34]. CXCR4 and CXCL12 are considered to be essential factors of allergic airway disease in the mouse [25, 35] and humans [36]. Thus, the increased circulating level of these chemokines that we have shown in our study may contribute to the process of recruitment of these cell types to the asthmatics' lungs. On the other side, the defective myelopoiesis and lymphopoiesis in parallel with lack of cardiovascular development in CXCL12 or CXCR4 (its receptor) may prove its role in homeostasis roles rather than inflammatory properties [19, 37, 38]; thus, increased circulatory level of CXCL12 in our asthma patients may be related to its role in remodeling of airways as an homeostatic chemokine rather than its other roles. Although previous reports indicated that the A allele is associated with higher CXCL12 expression [39], further studies pointed to the opposite side [40], and few studies claimed that there were no differences in the CXCL12 production by the A or G alleles [41, 42]. A more recent haplotype-based investigation [43] demonstrated that other polymorphisms in LD with the CXCL12 + 801G/A SNP, rather than the CXCL12 + 801G/A, are involved in control of the different transcription levels of CXCL12. Although the role of autoimmunity is not that much clear in the pathogenesis of asthma, SDF1-3′ G801A polymorphisms in Polish patients with systemic lupus erythematous which follow an autoimmune pattern are documented by Warchoł et al. [44]. As far as we sought to obtain more related studies in relation of asthma with this polymorphism we were unable but our studies on multiple sclerosis (Azin et al., [11]) and also unpublished data in type 1 diabetes as the two important diseases which similarly as asthma follow the pattern of autoimmunity revealed a close relation between autoimmunity and this polymorphism. Hence, there may possibly be an association between autoimmunity and the SDF-1*α* 3′A polymorphism that presents in autoimmune disorders. 

Nevertheless, our study was affected by some limitations. It should be considered that all of our study population (both control and asthma patients) were within Iranian population, and thus, the possibility of ethnicity as a confining factor could be excluded. However, it should be noted that different geographical and ethnic backgrounds of the individuals can influence the consequences of the association studies. Therefore, findings of the current study need to be examined and confirmed in other populations and ethnic groups. Moreover, further replication studies using a wider sample size and different population would be essential for further evaluation of the relationship between the CXCL12 genetic variations and the risk of asthma.

## Figures and Tables

**Table 1:** Subjects characteristics.

Variant	Controls	Asthma patients
Age	48 ± 12 years	51 ± 11 years
Gender: M/F	88/101 (189)	99/129 (228)
Frequent symptoms (cough, wheeze, and shortness of breath)	−	+
History of allergy	−	70.3%
Familial history of asthma	−	43.2%
FEV1 (predicted %)	87.3 ± 14	79.2 ± 21
FEV1/FVC	88.1 ± 9	82.3 ± 9
PEF (L/predicted %)	91.6 ± 21	74.6 ± 19
FEF.sub.25–75>	84.7 ± 17	62.9 ± 34
FVC (L/predicted %)	93.4 ± 8	79.9 ± 19
Eosinophils (106/L)	117 ± 96	612 ± 837
IgE (IU/mL)	24 ± 9	1,112 ± 1,082

FEV1: forced expiratory volume in 1 second, FVC: forced vital capacity, PEF: peak expiratory flow, and FEF.sub.25–75>: forced expiratory flow 25–75% or 25–50% (mean ± SD).

**Table 2:** Frequency of polymorphisms of SDF-1*α* (CXCL12) gene in asthma patients and controls.

	Patient *n* (%)	Control *n* (%)	OR	95% CI	*P* value
Genotype					
AA	18 (4.3%)	27 (6.5%)	2.541	1.17–5.51	*0.018
AG	190 (45.6%)	60 (14.4%)	0.144	0.77–0.268	*<0.0001
GG	20 (4.8%)	102 (24.5%)	4.021	2.21–7.29	*<0.0001

Alleles					
G	230 (27.6%)	264 (31.7%)	1		
A	226 (27.1%)	114 (13.7%)	2.276	1.70–3.03	*<0.0001

*Significant difference was observed.

**Table 3:** The results of analyzing data by Spearman's correlation coefficient test for relationship between protein level of SDF-1 and FEV1 percent (%) and between protein level of SDF-1 (pg/mL) and FEV/FVC (%) in patient group; significant difference (*P* < 0.0001) and inverse relationship (correlation coefficient: −1.00) were observed.

	Protein level of SDF-1			Correlation coefficient
	*N*	Mean ± SD	*P* value	
FEV1	103	79.2 ± 21	<0.0001	−1.00
FEV/FVC	103	82.3 ± 9	<0.0001	−1.00

**Table 4:** The mean rank of AA, AG, and GG genotypes for the protein level SDF-1, FEV1 percent, and FEV1/FVC percent in patient group. These data were produced by the Kruskal-Wallis test. There is a significant difference in all of them (*P* < 0.05 in all).

	Genotype	*N*	Mean rank	The Kruskal-Wallis test result
SDF-1	AA	12	71.38	*P* = 0.021
	AG	67	46.61	
	GG	24	56.79	

FEV1	AA	12	32.63	*P* = 0.021
	AG	67	57.19	
	GG	24	47.21	

FEV1/FVC	AA	12	32.63	*P* = 0.021
	AG	67	57.19	
	GG	24	47.21	
